# Difference in Presence and Number of CD83^+^ Dendritic Cells in Patients with Ulcerative Colitis and Crohn’s Disease

**DOI:** 10.1038/s41598-020-67149-5

**Published:** 2020-06-22

**Authors:** Bruna Rošić Despalatović, Marija Babić, Andre Bratanić, Ante Tonkić, Katarina Vilović

**Affiliations:** 1 ”J&J MEDICI” Polyclinic for Internal Medicine, Gynaecology and Psychiatry, Split, Croatia; 2 Medical School, University of Split, Split, Croatia; 3 Department of Gastroenterology and Hepatology, University Hospital Split, Split, Croatia; 4 Department of Pathology, University Hospital Split, Split, Croatia

**Keywords:** Medical research, Gastroenterology, Gastrointestinal diseases, Inflammatory bowel disease

## Abstract

Different pathophysiological models provide insight into the important role of CD83^+^ dendritic cells (DCs) in the pathogenesis of Crohn’s disease (CD) and ulcerative colitis (UC). There were 154 subjects included in this study: 60 with UC, 19 with CD and 75 in the control group. Colonic biopsy was performed in all subjects. Specimens were incubated with a primary anti-CD83 antibody. Intraepithelial DCs per 100 enterocytes were counted. The results were analysed according to demographic data, type of IBD and histological inflammation pattern. The odds ratio for CD83^+^ DCs=0 in the UC group was 3.4 times higher than that in the control group (OR = 3.4; 95% CI: 1.63–7.14; p = 0.001), and the odds ratio for CD83^+^ DCs ≥1 in the CD group was 5.3 times higher than that in the UC group (OR = 5.3; 95% CI: 1.4–20.2; p = 0.014). The odds ratio for CD83^+^ DCs=0 in the acute inflammation group was 2.7 times higher than that in the group without inflammation (OR = 2.7; 95% CI: 1.2–5.9; p = 0.011). In the group of patients with CD and acute inflammation (n = 11), there was only one subject without CD83^+^ DCs (p = 0,024). These results suggest an association of CD83^+^ DCs with the type of IBD and the histological inflammation pattern.

## Introduction

Inflammatory bowel diseases (IBDs) are chronic inflammatory diseases of the intestines that manifest as either Crohn’s disease (CD) or ulcerative colitis (UC). The pathogenesis of these diseases is not completely understood^1^. There is plenty of evidence that they originate from abnormal interactions between the intestinal microflora and the mucosal immune system in genetically susceptible individuals^2,3^. There is also increasing evidence that dendritic cells (DCs) play an important role in the induction and maintenance of chronic inflammation in IBD^4^. DCs belong to a family of antigen-presenting cells derived from bone marrow, and they are able to initiate the differentiation of naive T lymphocytes^5^. DCs are usually situated in areas of the body that make contact with the environment (skin and mucus membranes) and are thus continuously responding to the environment and recognizing and processing antigens^6,7^. These cells are also present in all vascular areas as partially mature DCs, where they recognize self as well as foreign antigens^5^. In addition, DCs receive signals from cytokines, intracellular components and lipids in the surrounding area. Integration of all these signals determines the cell’s immunologic response, which often results in the transformation of an antigen-recognizing cell into an antigen-presenting cell^5^. DCs represent the main population of antigen-presenting cells in the lamina propria in mice and rat models, as well as in humans^8–10^. DCs take the form of irregular MHC II-expressing cells^5^. In human models, DCs are present in the lamina propria and in lymphoid aggregates in the small intestines^11^. By flow cytometry of colonic and rectal specimens, an immature population of HLA^-^DR^+^lin^-^ DCs within the CD11c^+^ subpopulation was identified, and through maturation and growth in cell culture, the population obtained the phenotype of mature CD83^+^ DCs^12,13^. CD83 is a protein of the Ig gene superfamily originally identified in activated lymphocytes. Since then, CD83 has become an important marker for the identification of activated human DCs^12,13.^ In IBD patients, DCs seem to have an intrinsic abnormal responsiveness to antigens from the intestinal lumen.

The aim of this study was to determine the effect of subject demographic data (age and sex), type of IBD and histological inflammation pattern on the presence and number of mature intraepithelial CD83^+^ DCs in colonic biopsy samples.

## Methods

### Ethical issues

All subjects were informed about the goal, procedures, and course of this study. Before the onset, the study protocol was approved by the local Research Ethics Committee (Approval Class. 500–03/18-01/73; Approval NO 2181-147-01/06/m.S.-19-3, University Hospital Split, Croatia). All participants provided written informed consent, and all procedures were carried out in accordance with the 1964 Declaration of Helsinki and its later amendments, as well as the Good Clinical Practice guidelines from the International Conference on Harmonisation.

### Subjects

There were 154 subjects included in this study; all of them were older than 18 years and underwent colonoscopy during a two-year period from 2015 to 2017 at the Gastroenterology Clinic of “Polyclinic for Internal Medicine, Gynaecology and Psychiatry” J&J MEDICI. Among the included subjects were UC and CD subjects who were not receiving therapy, as well as healthy subjects in the control group. All subjects in the control group had no clinical symptoms and had normal endoscopic and histopathology findings. The diagnosis of UC was based on the ECCO criteria from 2008 to 2012 and the ECCO guidelines from 2018^14–16^. The diagnosis of CD was based on the ECCO guidelines from 2010, 2016, and 2018^16–18^. There were 60 subjects with UC and 19 subjects with CD, as well as 75 subjects in the control group.

### Endoscopy

Colonoscopy was performed with the VP 3500HD endoscopic video processor with an XL 4450 light source and EC530 and EC600 endoscopes (Fuji, Japan).

### Colonic biopsy sample analysis

Colonic biopsy samples of subjects with UC and CD were taken from sites of endoscopically visible disease. For UC, this was the descending colon, and for CD, it was the ascending colon. In the control group, biopsy samples were taken from the descending colon.

#### Histologic analysis

**Figure 1:**
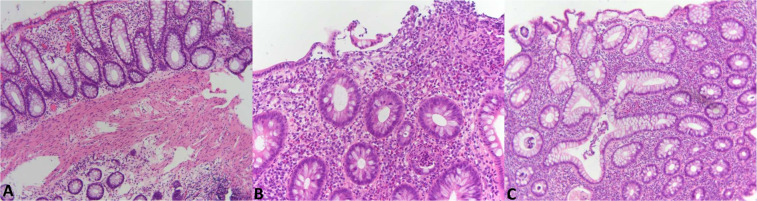
(**A**) Normal colonic mucosa: regular crypts and a normal lamina propria with lymphocytes, plasma cells, and eosinophils. **(B)** Chronic active inflammation in colitis: neutrophil-mediated epithelial injury in the form of crypt abscess and epithelial degenerative changes. **(C**) Chronic inflammation in colitis: crypt architectural distortion and diffuse, mixed inflammation in the lamina propria.

Tissue specimens from the descending colon (UC and control samples) and ascending colon (CD samples) were fixed in 4% buffered formaldehyde for 24 hours and then washed in 0.1 M phosphate buffer. After dehydration in increasing alcohol concentrations and xylol, they were embedded in paraffin at 56 °C, cut into 4-micrometre slices and then attached to a positively charged subject slide (Superfrost Plus, Thermo Scientific, Waltham, MA, USA). After that, the specimens were stained with standard haematoxylin-eosin (HE) in an HE 600 automatic stainer (Ventana, Tucson, Arizona, USA). Stained specimens were analysed with an Olympus BX41 light microscope. According to the histopathologic results, the patients were divided into three groups: chronic inflammatory changes, acute inflammatory changes and normal histology. Normal colonic mucosa has regular crypts and a regular lamina propria with lymphocytes, plasma cells, and eosinophils. Histopathological markers of chronic inflammation in colitis are architecturally distorted crypts surrounded by diffuse inflammation in the lamina propria with plasmacytosis at the base of crypts. Chronic active inflammation in colitis includes neutrophil-mediated epithelial degenerative changes on a background of chronic colitis (Fig. 1)^19^.

#### Immunohistochemical analysis

**Figure 2:**
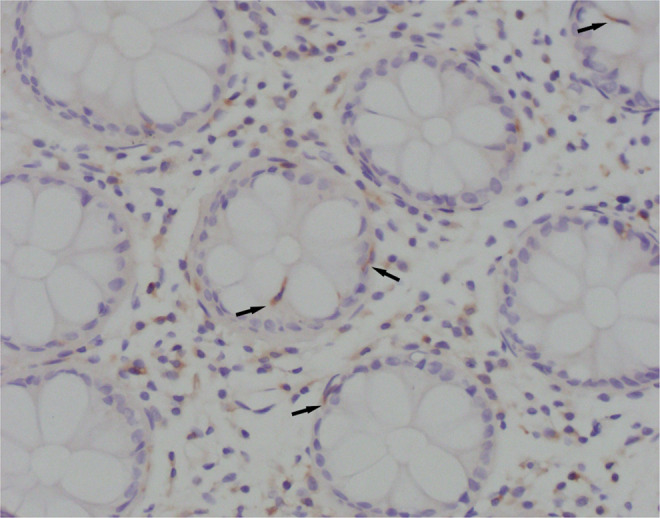
Light microscopy of CD83^+^ DCs (Olympus BX41; magnification ×40). Cytoplasmically brown-stained, irregularly shaped, CD83^+^ cells in close contact with the crypt epithelium.

For the immunohistochemical procedure, we used a primary anti-CD83 antibody (ab205343, Abcam, Cambridge, UK), a rabbit polyclonal antibody to CD83, suitable for IHC staining protocols^12,13,20^. The procedure was conducted by with a BenchMark ULTRA IHC/ISH staining system (Ventana, Tucson, Arizona, USA) with a positive control. After deparaffinization in xylol and rehydration in decreasing alcohol concentrations, specimen slices were incubated in EDTA buffer with pH 8,2 for 10–30 minutes. Endogenous peroxidase was inactivated by incubation in 3% H_2_O_2_ solution for 30 minutes at room temperature. After that, specimens were washed in phosphate-buffered saline (PBS) and incubated with the primary anti-CD83 antibody in a moist atmosphere for 32 minutes. After washing in PBS, specimens were incubated with reagents from the Ultraview Universal DAB Detection Kit (Ventana, Tucson, Arizona, USA). Stained specimens were analysed with an Olympus BX41 light microscope. We analysed the number of CD83-positive irregularly shaped cells in close contact with the crypt epithelium, which were morphologically and immunohistochemically different from lymphocytes (Fig. 2). In areas with the most intensive staining, intraepithelial CD83^+^ DCs were counted per 100 enterocytes.

### Statistic analysis

All data analysis was performed with SPSS 20. Statistical significance was set to p < 0,05, and all confidence intervals were given at 95%. As the Shapiro-Wilk test indicated statistically significant deviation from the normal distribution of all numeric variables, the median and interquartile ranges were used. Statistical significance of the differences in categorical demographic and clinical characteristics was calculated by the chi-square (χ^2^) test and Fisher’s exact test. Analysis of the statistical significance of differences in CD83^+^ DC number among the three study groups was performed with the Kruskal-Wallis test. Post hoc analysis was performed with the Mann-Whitney test. In our analysis, we also used binary logistic regression.

## Results

This study included 154 subjects older than 18 years. Among them, 60 (39%) had UC, 19 (12.3%) had CD, and 75 (48.7%) were in the control group. There were 85 (55%) males and 69 (45%) females. The median age of men was 42 years (Q1-Q3: 31–57 years; min-max: 15–79 years), and the median age of females was 42 years (Q1-Q3: 32–57 years; min-max: 18–79 years) (p = 0.676).

### Analysis according to disease type

Groups were adjusted according to age (p = 0.198) and sex (p = 0.737) (Table 1).

**Table 1:** Demographic characteristics according to the type of disease.

	Type of disease			p
	Control (n = 75)	UC (n = 60)	CD (n = 19)	
Age (years)	45 (32–59; 20–79)	42 (32–58.5; 15–79)	35 (27–52; 18–64)	0.198^a^
Sex				
Male	43 (57)	33 (55)	9 (47)	0.737^b^
Female	32 (43)	27 (45)	10 (53)	

Continuous data are presented as the median (interquartile range, min-max), and categorical data are presented as the number (percentage).

^a^Kruskal-Wallis test.

^b^ χ^2^ test.

The number of subjects with each histological inflammation pattern between the UC and CD groups did not differ significantly (p = 0.882) (Table 2).

**Table 2:** Histological inflammation pattern according to the type of the disease.

Histopathology inflammation pattern	Type of disease		p ^a^
	UC (n = 60)	CD (n = 19)	0.882
No inflammation	6 (10)	2 (10.5)	
Acute	36 (60)	11 (57.9)	
Chronic	18 (30)	6 (31.6)	

Categorical data are presented as numbers (percentages).

^a^ χ^2^ test.

**Figure 3:**
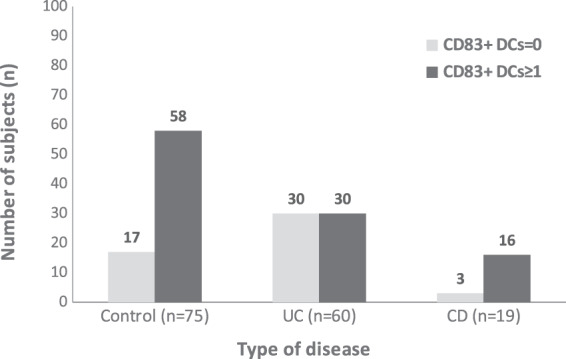
Distribution of CD83^+^ DCs (0, ≥1) according to the type of disease.

The presence of mature CD83^+^ DCs (0, ≥1) significantly differed among disease types (p = 0.001). A significant difference was found between the UC and control groups (p = 0.002) as well as between the UC and CD groups (p = 0.018). There was no difference between the control group and CD group (p = 0.513) (Fig. 3).

The odds ratio for CD83^+^ DCs=0/CD83^+^ DCs=1 in UC group was 3.4 times higher than that in control group (OR = 3.4; 95% CI: 1.63–7.14; p = 0.001). The odds ratio for CD83^+^ DCs ≥1/CD83^+^ DCs=0 in the CD group was 5.3 times higher than that in the UC group (OR = 5.3; 95% CI: 1.4–20.2; p = 0.014).

As we found an association between disease type and the presence of CD83^+^ DCs, we wanted to establish an association between the number of CD83^+^ DCs and disease type. There was a significant difference in CD83^+^ DC number among the studied groups (p < 0.001). The median CD83^+^ DC number was 0.5 times lower in the UC group of subjects than in the control group (p < 0.001) and 0.5 times lower in the UC group of subjects than in the CD group of subjects (p = 0.038), as calculated by post hoc analysis. There was no difference between the control group and CD group (p = 0.310) (Table 3).

**Table 3:** CD83^+^ DC number according to the type of disease.

	Type of disease			p
	Control (n = 75)	UC (n = 60)	CD (n = 19)	
**CD83**^**+**^ **DCs**	1.96 ± 2.16	0.82 ± 1,08	1.3 ± 1.16	
	1 (1–3;0–10)	0,5 (0–1;0–5)	1(1–1;0–4)	<0.001^a^
**CD83**^**+**^ **DCs**				
0	17 (22.7)	30 (50)	3 (15.8)	0.001^b^
1	23 (30.7)	18 (30)	12 (63.2)	
2	15 (20)	8 (13,3)	1 (5.3)	
3	11 (14.7)	2 (3,3)	1 (5.3)	
≥4	9 (11.9)	2 (3,3)	2 (10.5)	

Continuous data are presented as the mean ± standard deviation, median (interquartile range, min-max), and categorical data are presented as numbers (percentages).

^a^Kruskal-Wallis test.

^b^ χ^2^ test.

Regarding the number of CD83^+^ DCs, we established five categories (0, 1, 2, 3 and ≥4). We found an association of these categories with the type of disease (as represented by the control (healthy), UC, and CD groups) (p = 0.001). In the UC group, there were 30 subjects with CD83^+^ DCs = 0. The expected frequency was 19.5%. In the control group, there were 17 subjects with CD83^+^ DCs = 0, which was less than the expected frequency of 24.4%. The proportion of subjects with CD83^+^ DCs = 0 in the UC group was 2.2 times higher than that in the control group and 3.1 times higher than that in the CD group. In the CD group, there were 12 subjects with CD83^+^ DCs = 1. The expected frequency was 6.5%. The proportion of subjects with CD83^+^ DCs =1 in the CD group was 2 times higher than that in the control group and 2.1 times higher than that in the UC group (Table 3).

### Analysis according to histological inflammation pattern

The groups were adjusted according to age (p = 0.567) and sex (p = 0.582) (Table 4).

**Table 4:** Demographic characteristics according to histological inflammation pattern.

		Histological inflammation pattern			p
		No inflammation (n = 83)	Acute (n = 47)	Chronic (n = 24)	
Sex	Male	48 (58)	26 (55)	11 (46)	0.582^b^
	Female	35 (42)	21 (46)	13 (54)	
Age (years)		45 (32–60; 20–79)	40 (31–57; 15–78)	39 (32–50; 21–79)	0.567^a^

Categorical data are presented as numbers (percentages), and continuous data are presented as medians (interquartile ranges, min-max).

^a^Kruskal-Wallis test.

^b^ χ^2^ test.

**Figure 4:**
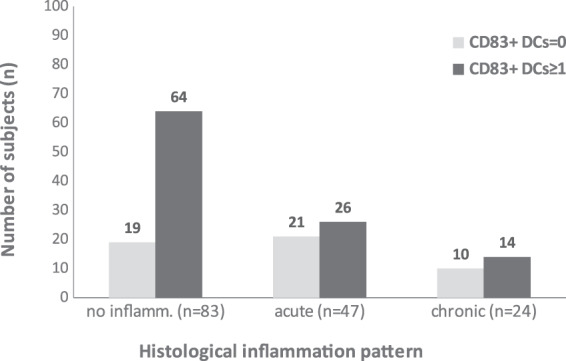
Distribution of CD83^+^ DCs (0, ≥1) according to histological inflammation pattern.

The presence of mature CD83^+^ DCs (0, ≥1) differed significantly among groups with different histopathologic patterns of inflammation (no inflammation, acute or chronic) (p = 0.022). There was a difference between the group with acute inflammation and the group with no inflammation (p = 0.017). There was no difference between the acute inflammation and chronic inflammation groups (p = 1) or between the chronic inflammation group and the group with no inflammation (Fig. 4).

The odds ratio for CD83^+^ DCs=0 in the acute inflammation group was 2.7 times higher than that in the group with no inflammation (OR = 2.7; 95% CI: 1.2–5.9; p = 0.011).

After we found an association between the presence of CD83^+^ DCs and the histological inflammation pattern (no inflammation, acute or chronic), we also found an association between the number of CD83^+^ DCs and the histological inflammation pattern (p = 0,001) (Table 5). A difference in the number of CD83^+^ DCs was found between the acute inflammation group and the group with no inflammation (p = 0.02) as well as between the chronic inflammation group and the group with no inflammation (p = 0.036) by post hoc analysis.

**Table 5:** CD83^+^ DC number according to histological inflammation pattern.

	Histological inflammation pattern			p
	No inflammation (n = 83)	Acute (n = 47)	Chronic (n = 24)	
CD83^+^ DCs	1 (1–3;0–10)	1 (0–1;0–4)	1 (0–1;0–4)	0.001^a^
	1.93 ± 2.1	0.85 ± 1	0.88 ± 0.99	
CD83^+^ DCs				0.034^b^
0	19 (22.9)	21 (44.7)	10 (41.7)	
1	26 (31.3)	18 (38.3)	9 (37.5)	
2	16 (19.3)	4 (8.5)	4 (16.7)	
3	12 (14.5)	2 (4.2)	0 (0)	
≥4	10 (12)	2 (4.3)	1 (4.2)	

Continuous data are presented as the median (interquartile range, min-max) and mean ± standard deviation, and categorical data are presented as numbers (percentages).

^a^Kruskal-Wallis test.

^b^ χ^2^ test.

When we categorized subjects according to the number of CD83^+^ DCs (0, 1, 2, 3, or ≥4), we found a statistically significant association between these categories and the histological inflammation pattern (p = 0.034). In the group with no inflammation, there were 19 subjects with CD83^+^ DCs=0; the expected frequency was 27%. The proportion of subjects with CD83^+^ DCs=0 in the group with no inflammation was 1.95 times lower than that in the acute inflammation group and 1.82 times lower than that in the chronic inflammation group. In the group with CD83^+^ DCs=3, there were 12 subjects, and the expected frequency was 7%. The proportion of subjects with CD83^+^ DCs=3 in the group with no inflammation was 3.4 times higher than that in the acute inflammation group (Table 5).

Finally, we analysed the presence of CD83^+^ DCs (0, ≥1) according to the type of disease combined with the histological pattern of inflammation (Table 6). Since we did not prove a statistically significant difference in the presence of CD83^+^ DCs (0, ≥1) between the CD and UC groups with chronic inflammation (Fisher’s exact test: p0 = 0.340), we combined these two groups together into one group for the χ^2^ test. In the group of subjects with CD and acute inflammation (n = 11), there was only one subject without CD83^+^ DCs (p = 0.024) (Table 6).

**Table 6:** Presence of CD83^+^ DCs (0, ≥1) according to the type of disease combined with the histological pattern of inflammation.

Studied groups (combined)	Total n = 71	CD83^+^ DCs		p
		0 n = 31	≥1 n = 41	0.024^a^
UC, acute inflammation	36 (50.6)	20 (64.5)	16 (40)	
CD, acute inflammation	11 (15.5)	1 (3.3)	10 (25)	
UC, chronic inflammation	18 (25.4)	8 (29)	9 (22.5)	
CD, chronic inflammation	6 (8.5)	1 (3.2)	5 (12.5)	

Categorical data are presented as numbers (percentages).

^a^χ^2^ test.

The χ^2^ test was used to calculate the presence of CD83 + DCs according to the following groups: UC and acute inflammation; CD and acute inflammation; UC and chronic inflammation, and CD and chronic inflammation.

## Discussion

In our study, the presence and number of mature CD83^+^ DCs in colonic biopsy specimens of UC and CD subjects who had not received therapy and of control subjects were analysed.

We found a significant difference in the presence of CD83^+^ DCs among different types of inflammatory bowel diseases. There were significantly fewer subjects with CD83^+^ DCs ≥1 in the UC group than in the CD group. These cells were significantly more frequent in CD subjects than in UC subjects, regardless of sex and age. Very few studies have analysed the presence of mature DCs in different types of inflammatory bowel disease. Radwan *et al*. compared the number of mature DCs in the intestinal mucosa in different inflammatory bowel diseases with those in healthy subjects. A significantly increased presence of mature DCs was found in both disease groups (UC and CD) compared with that in the control group^21^. Middel *et al*. also found an increased presence of CD83^+^ DCs in the tissues of CD and UC patients^22^. In previously published studies on CD, there was also an increased presence of DCs in mucosal tissue, and these DCs expressed costimulatory molecules such as CD40, CD80, CD83 and CD86^22,23^. On the other hand, Baumgart *et al*. reported low expression of the costimulatory molecule CD86 on DCs of IBD patients, while CD83 expression was absent^24^. However, it must be stressed that their analysis was performed on peripheral blood samples. There were identical results in the studies of Velde *et al*. and Middel *et al*.^13,22^.

Our study also analysed the number of mature CD83^+^ DCs between different types of inflammatory bowel diseases. We showed that there was a significantly smaller number of CD83^+^ DCs in UC subjects than in CD or in control subjects. Bates *et al*. reported results from a mouse colitis model study in which CD83^+^ DC function had been studied^25^. Their results showed that loss of CD83 in DCs led to worsening of inflammation in the colitis model^25^. They also confirmed that DCs isolated from the lamina propria of mice with colitis had significantly decreased expression of the maturation marker CD83^25^. This mouse model could explain how DCs in immunologic reservoirs, such as the intestinal lamina propria, prevent excessive inflammation. In contrast to our study, Kawashima *et al*., found an increased number of CD83^+^ DCs isolated from cell culture of surgical specimens from UC patients via immunofluorescence staining, primarily in lymphoid aggregates from specimens with histologically active disease^26^. An increased number of CD83^+^ DCs was found by Baumgart *et al*., this time isolated from peripheral blood by immunocytochemistry^27^. Bell *et al*., using multicolour flow cytometry, did not find any significant evidence for an increase in DC number in inflamed colonic tissue in IBD patients compared to healthy controls, although the median values were higher.^12^

Our study also showed that there was a significant difference in CD83^+^ DC presence and number among subjects with different histological inflammation patterns. Middell *et al*. found an increased number of CD83^+^ DCs in specimens from locations with active inflammation compared with specimens from locations without inflammation in the same patient^22^. In most of studies so far, the number of DCs correlated with the severity and extent of the inflammatory process^21,24,28^.

Our results showed that the number of UC subjects who had CD83^+^ DCs was similar in those subjects who had active inflammation (44%) and those who did not (56%), while the proportion of CD subjects with acute inflammation that had these cells was significantly higher (91%). Previous studies on DCs in IBD patients who have not received therapy included only CD subjects. Vestege *et al*. did not find any change in the CD83^+^ DC number in CD patients in their study, while Velde *et al*. confirmed an increased prevalence of CD83^+^ DCs in the tissue of CD patients^13,29^. All previous studies on UC included only patients receiving immunosuppressive therapy.

Considering our results, it can be concluded that the sex and age of IBD subjects do not affect the presence or number of CD83^+^ DCs. What actually affects CD83^+^ DC presence and number in these subjects is the type of the disease itself and the histological inflammation pattern, with these cells being most frequent in subjects with CD and acute inflammation.

## Data Availability

We disclose no restrictions on the availability of data, materials and associated protocols.
